# Transtracheal Wash Fluid Collection and Analysis in Healthy Goats

**DOI:** 10.1111/jvim.70211

**Published:** 2025-09-17

**Authors:** Bridget J. Savitske, Richard J. Dulli, Narayan C. Paul, Kevin E. Washburn

**Affiliations:** ^1^ Department of Large Animal Clinical Sciences College of Veterinary Medicine & Biomedical Sciences, Texas A&M University College Station Texas USA; ^2^ Department of Pathobiology College of Veterinary Medicine & Biomedical Sciences, Texas A&M University College Station Texas USA; ^3^ Texas A&M Veterinary Medical Diagnostic Laboratory College Station Texas USA

**Keywords:** bacterial culture, caprine, cytology, respiratory

## Abstract

**Background:**

Transtracheal wash (TTW) is frequently used to characterize respiratory disease in companion animals, cattle, and horses, but no description of TTW methods or fluid analysis in healthy goats is available.

**Hypothesis/Objectives:**

Describe a technique for collecting TTW fluid (TTWF) from healthy goats and describe the nucleated cell populations within and bacterial populations isolated from TTWF in healthy goats.

**Animals:**

Thirty‐three healthy university‐owned Boer does.

**Methods:**

Prospective descriptive study. Percutaneous TTW was performed in sedated healthy goats. Retrieved TTWF from each goat was analyzed for cytologic findings, and aerobic and anaerobic microbiologic cultures were performed.

**Results:**

Aliquot recovery volume averaged 10.8%. Four doses were excluded from summary statistics for TTWF cytology and culture. In 29 TTWF samples, macrophages were the predominant cell population (40%–92%) followed by small lymphocytes (3%–39%) and neutrophils (2%–32%). Cultures of most samples yielded mixed bacterial growth (62.1%, 18/29). *Bibersteinia trehalosi* was the most frequently identified bacterial species from pure culture (17.2%, 5/29). No association was found between the cytologic presence of bacteria and bacterial culture results (*p* = 0.63).

**Conclusions and Clinical Importance:**

The described TTW method is a minimally invasive diagnostic procedure in goats that can be performed in a hospital or field setting with commercially available materials. Our results describe the cytologic and bacterial populations identified within TTWF of healthy goats. Cytology results do not predict bacterial growth in healthy goats. Bacterial genera that are routinely associated with bronchopneumonia in goats also are present in health.

**Abbreviations**
BAL: bronchoalveolar lavage
CBC: complete blood count
EDTA: ethylenediaminetetraacetic acid
MALDI‐TOF MS: matrix‐assisted laser desorption/ionization time‐of‐flight mass spectrometry
MNGC: multinucleated giant cell
PCR: polymerase chain reaction
RI: reference interval
TNCC: total nucleated cell count
TTW: transtracheal wash
TTWF: transtracheal wash fluid

## 1 Introduction

Respiratory disease is one of the most frequent and costly diseases impacting each sector of the livestock industry in North America [1]. Losses are accrued from decreased feed efficiency, mortality, unfavorable carcass traits, and the cost of treatment itself [2, 3]. Marshaling techniques to improve accuracy and speed in diagnosing the causes of lower airway disease will help address the growing problem of antimicrobial resistance in common respiratory pathogens [4, 5, 6, 7]. Nasal swabs can become heavily contaminated and may not represent the bacterial communities within the lower airway, thus complicating clinical interpretation [8]. Both false positive and false negative diagnoses of pneumonia are encountered with radiographic and ultrasonographic imaging, depending on technique, stage of disease, type of inflammation, individual interpretation, as well as animal age and size [9, 10, 11, 12, 13, 14, 15]. Bronchoalveolar lavage (BAL) offers useful cytologic information about the lower airway and, because it is considered a rapid and economical diagnostic test when performed by blind technique (rather than endoscope‐guided), is frequently performed in cattle for bacterial culture [16, 17]. Segmental disease, as lower airway infection often presents, and contamination by upper airway commensal organisms pose limitations to BAL that could result in inaccurate interpretations. For these reasons, although TTW is not infallible, it is often preferred over BAL when sampling for infectious causes of lower airway disease [18, 19, 20].

Although TTW in goats occasionally is mentioned in literature, the techniques for the procedure are not well described [21, 22, 23, 24]. Transtracheal wash fluid is utilized routinely for evaluation of lower airway disease in horses and cattle of all ages [25, 26, 27, 28, 29]. There are also reports of its utility in other large animal species including llamas [30, 31], alpacas [32], dromedary camels [32, 33], and production hogs [34]. Transtracheal wash generally is considered a safe procedure, with rare or mild post‐procedural complications [18].

The lack of investigation of the technique has left a gap in knowledge pertaining to the cytologic and microbiologic characteristics of TTW fluid (TTWF) in healthy goats. Because different leukocyte and bacterial populations are anticipated at different levels of the airway, a baseline must be constructed to allow for meaningful interpretation of results [25, 29]. It is also critical that cytology and culture be interpreted in light of each other and with clinical presentation. Establishing a practical and appropriate method for performing TTW in goats would enhance the ability of practitioners to diagnose, monitor, and select appropriate treatment for their caprine patients. Notwithstanding, interpretation of TTWF analysis results in diseased goats requires characterization and description of TTWF analysis in a population of healthy goats. Thus, our objectives were to describe a technique for collecting TTWF from goats and to describe the cytologic population distribution and the frequency of distribution of bacteria in TTWF collected in healthy goats.

## 2 Materials and Methods

### 2.1 Ethics Statement

This study was approved by the Texas A&M Institutional Animal Care and Use Committee.

### 2.2 Study Population

The study population was comprised of 33 healthy Boer does from a university‐owned closed teaching herd. The number of goats was selected based upon funding and available research animals. All goats were healthy Boer‐cross does ranging from 6 months to 6 years of age, and identification was based on ear tags. No doe was pregnant or had weaned any kids at least 2 months before the initiation of the study. All does had received a clostridial vaccine within the previous 12 months, according to routine herd management practices. Each goat was evaluated by thorough physical examination performed by the author (BJS), including lung and tracheal auscultation. Thoracic ultrasonography was performed using a linear rectal transducer, according to methods described to assess pulmonary disease in calves and goats [35, 36, 37, 38]. To minimize subjectivity with ultrasonographic interpretation, each animal was assigned an ultrasound score using similar criteria to a previously described system for calves [39]. Modifications to this scoring system were based upon the authors' (BJS, KEW) clinical experience and literature pertaining to ultrasonographic findings of both healthy and diseased lung tissue [40, 41, 42]. Briefly, animals were scored on a scale of 0–5 (0—absent to occasional comet‐tail artifacts [B‐lines]; 1—frequent to diffuse comet‐tail artifacts; 2—≥ 1 areas of consolidation measuring ≤ 1 cm [dorsal‐to‐ventral plane]; 3—≥ 1 areas of consolidation measuring 1–3 cm; 4—one region of consolidation measuring > 3 cm; 5—regional consolidation occupying ≥ 2 lung lobes). A CBC, which included plasma fibrinogen concentration measured by heat precipitation, was performed on each animal to assess for evidence of active infectious or inflammatory processes. Fecal samples were collected rectally, and each animal was screened for lungworms using the modified Baermann technique. Criteria for exclusion included ultrasound score ≥ 1; body condition score ≤ 2/5; FAMACHA score ≥ 4/5; a history of clinical signs of respiratory disease within 3 months of the study; treatment with an antimicrobial within the previous 60 days; neutrophilia > 7.2 × 10^3^ cells/μL with a concurrent left shift or toxic changes [43]; neutropenia < 1.2 × 10^3^ cells/μL [43]; or the presence of lungworm larvae in the feces. To replicate a typical patient population, the animals were housed outdoors, provided ad libitum access to Coastal Bermuda grass pasture and water, and supplemented with approximately 1% body weight of a 15% protein goat‐specific feed once daily. All does had been acclimated to a new pasture for a minimum of 7 days before sampling.

### 2.3 Transtracheal Wash Procedure

**FIGURE 1:**
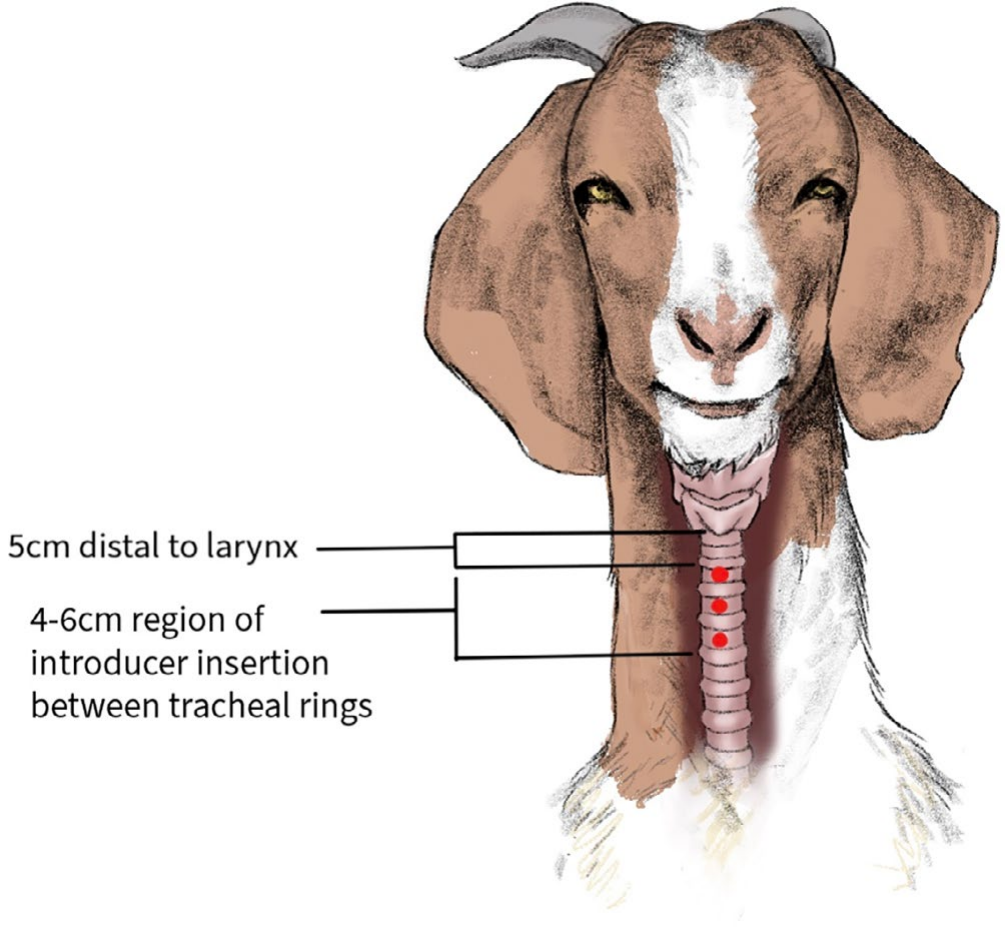
Schematic of the head and neck of an adult Boer doe demonstrating the area of preparation and possible sites of puncture by the introducer in between the tracheal rings.

Does were sedated with midazolam hydrochloride IV (0.2 mg/kg; Avet Pharmaceuticals Inc., East Brunswick, NJ) and butorphanol tartrate IV (0.05 mg/kg; Torbugesic, Fort Dodge Animal Health, Fort Dodge, IA) and restrained in a standing position with the neck extended. A 4 × 6 cm area approximately 5 cm distal to the larynx was clipped and antiseptically prepared with 3 repetitions of alternating 4% chlorhexidine gluconate scrub (Hibiclens, Mölnlycke Health Care, Norcross, GA) and 70% isopropyl alcohol. The prepared region was palpated, and a space between 2 tracheal rings was identified as the intended site of entry. Regional anesthesia was performed by SC injection of 2 mL of 2% lidocaine (LidoJect, 20 mg/mL, Henry Schein Animal Health, Dublin, OH) using a 20‐gauge needle. Using a #15 surgical blade (C.R. Bard Inc., Murray Hill, NJ), a 1‐cm skin incision was created over the intended site of tracheal entry to expose the subcutaneous tissue and muscle. The remainder of the TTW procedure utilized the components within the Mila Large Animal Transtracheal Wash Kit (Mila International Inc., Florence, KY). A 10‐gauge introducer was inserted through the incision and between the tracheal rings into the tracheal lumen (Figure 1). After confirmation of placement of the introducer into the tracheal lumen, the stylet was retracted, and the sheath advanced until securely in place. A 12‐gauge × 70‐cm irrigating catheter was introduced through the introducer sheath. Using the catheter as an indicator of length, the irrigating catheter was advanced to the region estimated to be just beyond the thoracic inlet, and then the stylet was removed. An aliquot of 0.9% sterile saline (0.4 mL/kg) was instilled and immediately aspirated. If an insufficient volume was retrieved to perform both culture and cytology, a second aliquot of saline (0.2 mL/kg) was instilled and immediately aspirated. Upon completion of fluid retrieval, the irrigating catheter was removed, followed by the introducer sheath. The area was cleaned with alcohol on removal of the catheters, and the site left open to heal. Upon retrieval, the volume of lavage fluid was recorded and equally divided into a sterile no‐additive sample tube (for microbiologic culture) and an ethylenediamine tetraacetic acid (EDTA) sample tube (for cytologic analysis) and refrigerated at 4°C until processing, which occurred within 4 h of acquisition. Each animal was examined by the author (BJS) daily for 1 week after the procedure. Any abnormalities or adverse effects were recorded, and treatment was implemented as required.

### 2.4 Cytology

Nucleated cells were counted in the TTWF using the Z1 particle counter (Beckman Coulter; Brea, CA). A cytocentrifuge slide was prepared with 100 μL of TTWF and stained with a modified Wright stain, and then examined by the author (RJD). A 400‐cell differential count of leukocytes and other clinically relevant cytologic findings was recorded.

### 2.5 Isolation and Identification of Bacteria

The TTWF samples were streaked onto 5% sheep blood agar, phenylethylalcohol agar, and tergitol agar (Hardy Diagnostics, Santa Maria, CA) plates and incubated in aerobic and anaerobic conditions at 37°C for 48 h. All of the culture plates were read at 24 and 48 h for isolation of bacteria. Initial identification of bacteria was based on colony morphology on plates, and different biochemical test results including oxidase, catalase, coagulase, indole, carbohydrate fermentation, and gram staining. Identification of different bacteria up to genus or species level was based on matrix‐assisted laser desorption‐ionization time of flight mass spectrometry (MALDI‐TOF MS; Bruker Daltonics, Bremen, Germany) score results. A score of 2.3–3.0 was considered a highly probable species identification, and a score of 2.0–2.299 was considered a secure genus identification and probable species identification. Final identification of all bacterial isolates was based on the agreement between biochemical and MALDI‐TOF MS results. When mixed bacterial colonies with no predominant colony types were present on bacterial culture plates, these were reported as “mixed bacterial growth”.

### 2.6 Fungal Culture

The TTWF samples were streaked onto Sabouraud dextrose agar, potato dextrose agar, and 5% sheep blood agar plates for isolation of fungi. Plates were incubated for 14 days at room temperature and examined for any fungal growth. Fungal identification was based on colony morphology and microscopic observation using lactophenol cotton blue staining.

### 2.7 Statistical Analysis

Reference intervals (RIs) for the TTWF cytology results were determined using MedCalc Statistical Software version 22.013 (MedCalc Software Ltd., Ostend, Belgium; https://www.medcalc.org; 2023) according to American Society for Veterinary Clinical Pathology (ASVCP) Quality Assurance and Laboratory Standards Committee (QALS) guidelines for the determination of reference intervals in veterinary species recommendations when 20–40 samples are available [44]. In brief, the results of the 400‐cell differentials were evaluated by the D'Agostino‐Pearson test for normality. Reference intervals with 90% confidence intervals were determined by the parametric method if normally distributed, or by the robust method with bootstrapping if not. Outliers were assessed by Tukey's test. Fisher's exact test was used to determine associations between the cytologic presence of bacteria and bacterial culture outcome. A *p*‐value < 0.05 was considered significant. Additionally, median, range, and mean (when normally distributed) were reported for each cell population.

## 3 Results

### 3.1 Health Assessments

Thirty‐three does were included for sample collection. All does were negative for all species of lungworm on the modified Baermann test, but gastrointestinal nematode larvae or eggs were noted in 22/33 animals (66.6%). Body weights ranged from 20.5 to 77.3 kg (median, 64.5 kg) and body condition scores ranged from 2.5 to 4 (median, 3). The FAMACHA scores ranged from 1 to 3 (median, 3). Numerous CBCs had mild to moderate eosinophilia (RI, 0–700 cells/μL) and mild to moderate basophilia (RI, 0–100 cells/μL). Five animals had reactive lymphocytes on blood smear. One animal had mild hyperfibrinogenemia (500 mg/dL; RI 100–400) with an otherwise normal CBC and cell morphology. Four blood samples were extensively clotted, and thus these CBCs were not interpretable. Total white blood cell counts with leukocyte differential, hematocrit, plasma protein, and fibrinogen data are presented in Table S1. All 33 tested goats were included for TTW because the reported eosinophilia, basophilia, and mild hyperfibrinogenemia were considered clinically unimportant, and the four goats for which the CBC was not reported met the remainder of the inclusion criteria.

### 3.2 Transtracheal Wash Fluid Cytologic Findings

Thirty‐three TTWF samples were collected. Recovery volume of TTWF averaged 10.8% (median, 10.7%; range, 4.5%–19.8%).

**FIGURE 2:**
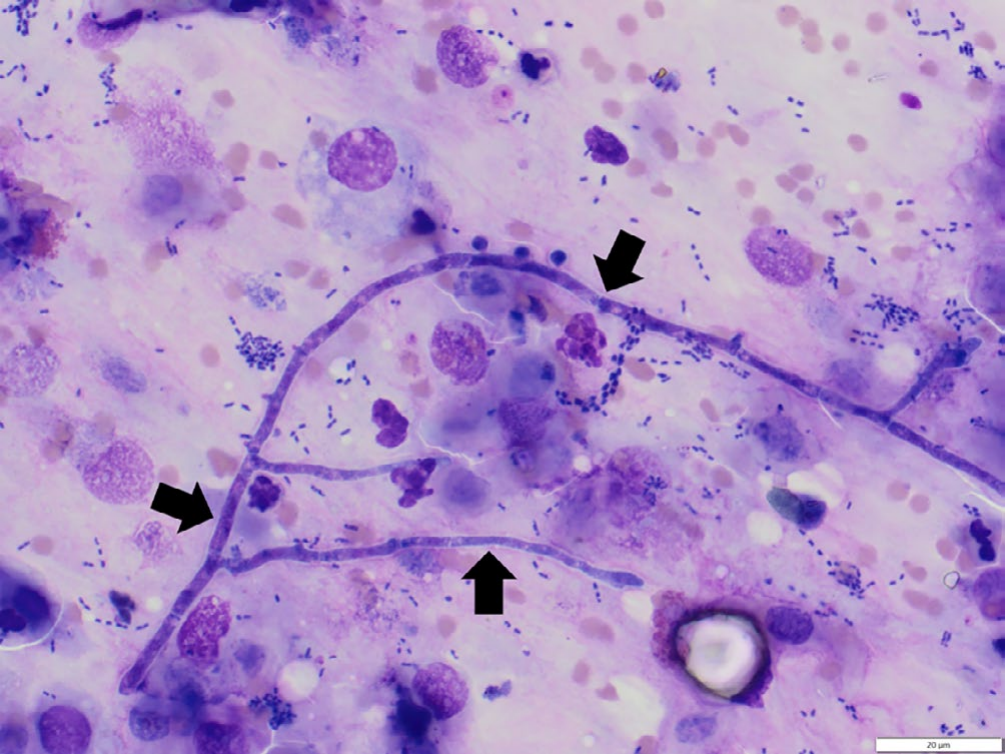
Cytospin preparation of transtracheal wash fluid cytology from a goat excluded from analysis despite normal clinical presentation. There are abundant intracellular and extracellular bacilli along with occasional branching fungal hyphae (arrows). Degenerate neutrophils were 72% of the inflammatory cell population. Slide prepared with modified Wright Giemsa stain at 60× objective. Scale bar = 20 μm.

**FIGURE 3:**
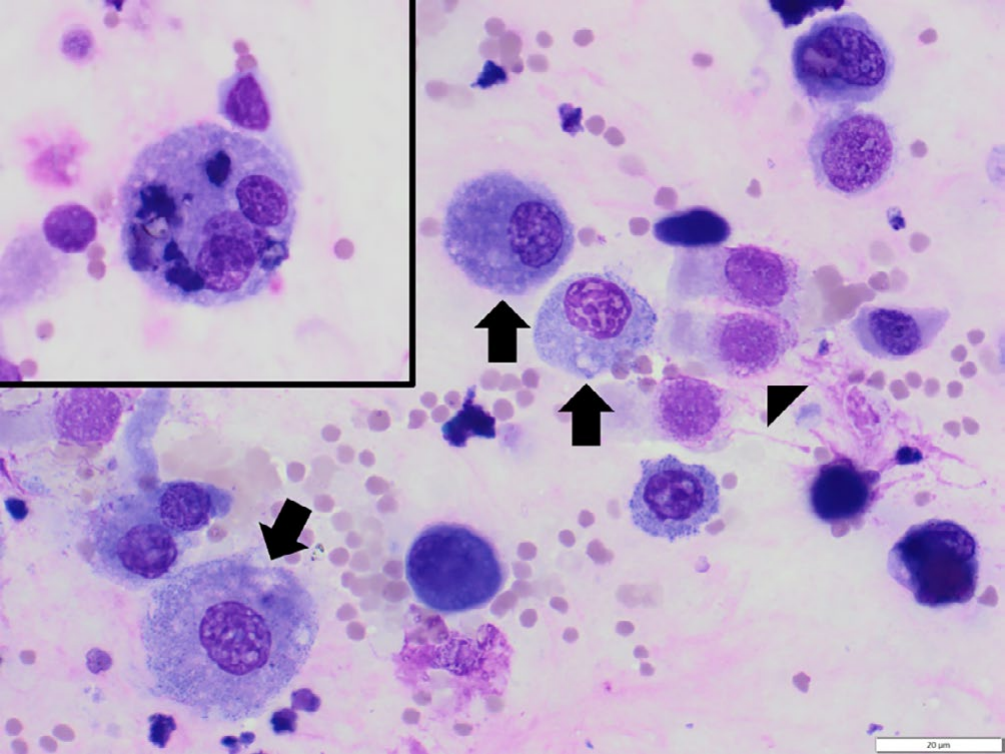
Cytospin preparation of transtracheal wash fluid cytology from a healthy goat. As seen in most samples, the slide shows a macrophage‐predominant cellular population (arrows) with lower numbers of small lymphocytes and neutrophils and rare eosinophils and mast cells. There are variably preserved columnar respiratory epithelial cells (arrowhead) and one binucleated macrophage (inset), also seen in most samples. Slide prepared with Modified Wright Giemsa stain at 60× objective. Scale bar = 20 μm.

Of the 33, one was excluded from summary statistics because of insufficient cellularity despite adequate recovery volume (non‐diagnostic sample). Another three TTWF samples with the highest neutrophil percentages were excluded because of the presence of fungal hyphae (37.25% neutrophils), intracellular bacteria (56.5% neutrophils), or both (72.5% neutrophils) in the samples (Figure 2). In the remaining 29 samples, macrophages were the predominant cell population along with variable numbers of small lymphocytes and neutrophils (Figure 3). Eosinophils and mast cells were rare to absent in all samples. Summary statistics and RIs based on the remaining 29 samples are shown in Table 1. The total nucleated cell count (TNCC) median and mean were 1806 cells/μL and 2393 cells/μL, respectively (range, 359–8827 cells/μL). Variable numbers of erythrocytes were seen in all samples (median, 7878 RBC/μL; mean, 36 959 RBC/μL; range, 519–478 453 RBC/μL). Additional microscopic findings included variable numbers of ciliated columnar respiratory epithelial cells (96.5%, 28/29; Figure 3), rare (< 1%) binucleated macrophages or multinucleated giant cells (MNGCs) or both (86.2%, 25/29; Figure 3), rare (< 1/1000× field) extracellular bacteria (31.0%, 9/29), and non‐infectious fungal spores, pollen, or refractile crystalline material presumed to represent inhalation of debris when intracellular or environmental contamination when extracellular (79.3%, 23/29). Neutrophil morphology was well‐preserved. Occasional degenerative change alone was not considered suggestive of infection because of the unreliability of this finding in respiratory wash cytology [45]. No lungworm larvae or ova were seen. Findings are summarized in Tables S2 and S3.

**TABLE 1:** Leukocyte percent reference intervals derived from 400‐cell differential of transtracheal wash fluid (TTWF) in 29 healthy Boer does.

	Mean	Median	Range	LL of RI (90% CI)	UL of RI (90% CI)
Neut^a^	14	12	2–32	0 (0–4)	28 (24–32)
Lymph^b^	N/A	11	3–39	0 (0–0)	28 (21–34)
Mφ^a^	73	73	40–92	49 (42–56)	98 (91–100)
Eos^b^	N/A	0	0–11	0 (0–0)	1 (1–1)
Mast^b^	N/A	0	0–13	0 (0–0)	2 (1–2)

Abbreviations: Eos, eosinophils; LL, lower limit; Lymph, lymphocytes; Mφ, macrophages; Mast, mast cells; N/A, not applicable; Neut, neutrophils; RI, reference interval; UL, upper limit.

^a^ Results were normally distributed based on D'Agostino‐Pearson test. Reference intervals calculated by the parametric method (ASVCP guidelines reference).

^b^ Results were not normally distributed based on D'Agostino–Pearson test. Means are not reported and RIs are determined by the robust method with 90% confidence intervals from bootstrapping (ASVCP guidelines reference).

### 3.3 Transtracheal Wash Fluid Microbiologic Findings

The culture results of the included 29 TTWF samples are presented in Table S4. One aliquot of saline was submitted as a negative control, and no bacterial or fungal growth was observed. Overall, mixed bacterial growth was observed in the majority of samples (62.1%, 18/29). Bacteria identified by pure culture were *Bibersteinia trehalosi* (17.2%, 5/29), *Enterobacter* spp. (10.3%, 3/29), *Fusobacterium* spp. (6.8%, 2/29), *Mannheimia* spp. (6.8%, 2/29), *Escherichia coli* (3.4%, 1/29), and *Rhodococcus equi* (3.4%, 1/29). No bacterial growth was obtained in 17.2% (5/29) of samples, and 12.5% (4/29) grew fungi. Fungal isolates were not characterized further. Based on Fisher's exact test, the cytologic presence of bacteria was not significantly associated with bacterial culture outcome (*p* = 0.63).

### 3.4 Post‐Procedure Observations

Because of small tracheal size and a smaller space between tracheal rings, the introducer placement was subjectively more challenging in smaller does. These does were given flunixin meglumine IV (2.2 mg/kg; Prevail, MWI Veterinary Supply Co., Boise, Idaho) after sample acquisition. All does developed moderate edema at the transtracheal wash site, and five does developed a small subcutaneous abscess at the transtracheal wash site. Neither the edema nor abscessation impacted quality of life, including breathing, neck movement, and swallowing. The edema and abscesses were monitored closely, conservatively managed, and resolved within 4 weeks of the TTW procedure. No doe, including those excluded based on cytologic evidence of infection, developed respiratory signs within the 1‐month follow‐up period.

## 4 Discussion

Transtracheal wash is a commonly used clinical tool for the diagnosis and research of respiratory disease in small companion animals, cattle, and horses. To date, limited information about the use of TTW in goats is available [21, 22, 23, 24]. Using inexpensive and readily available materials, TTW was efficient and generally well tolerated. The technique yielded diagnostic quality samples in most cases, with only one sample of 33 excluded for low cellularity.

### 4.1 Transtracheal Wash Fluid Cytologic Findings

The TTWF cytologic findings generally aligned with our expectations based upon experience with horses and cattle and the available veterinary literature [25, 29, 45, 46, 47]. In terms of inflammatory cell proportions, mononuclear cells (macrophages and lymphocytes) predominated in most does along with fewer neutrophils and only rare eosinophils and mast cells. A relatively high degree of variability was found in the frequency of neutrophils among animals, but this observation is consistent with what has been reported in other large animal species. For example, in horses, up to 20% neutrophils is generally considered normal in TTWF [45], but studies in horses have repeatedly found higher numbers of TTWF neutrophils in some clinically normal animals [25, 46, 47]. The frequent presence of intra‐ and extracellular fungal spores and silica crystals is also commonly seen in horses, and likely reflects inhalation of dust and debris from their feed and environment [29, 45, 46]. This finding also explains the presence of binucleated macrophages and MNGCs in most samples, which, as in horses, likely represents a normal inflammatory response to chronic inhalation of foreign material (Figure 3) [45].

### 4.2 Transtracheal Wash Fluid Microbiologic Findings

No significant association was found between cytologic visualization of bacteria and positive culture results. This finding was not altogether surprising because culture is more sensitive than cytology in the detection of bacterial presence [48]. Besides contamination, bacteria can be cultured despite the lack of cytologic visualization if bacterial numbers were low in the TTWF sample or the organisms did not withstand the slide preparation. Future research should investigate factors associated with cytologic and microbiologic discordance, such as animal size, aliquot volume, degree of bacterial growth, timing of sampling within the disease course, and processing delay.

Because the trachea is not sterile, it is not unexpected that most cultures yielded mixed bacterial growth [49]. The bacteria identified by pure culture, however, were consistent with organisms identified by tracheal brushing samples in healthy sheep in one report but dissimilar to those of tracheobronchial aspirates in another [50, 51]. Interestingly, in one report, larger proportions of genera belonging to the *Pasteurellaceae* family (*Mannheimia*, *Pasteurella*, *Bibersteinia*) were found in tracheal samples compared with samples obtained from beyond the tracheal bifurcation [49]. It was unexpected that our culture results did not identify the caprine airway commensal bacteria *Pasteurella multocida*, which could be attributed to its being confined to the nasopharynx in healthy animals [52, 53]. Because of considerable differences in sample size, airway sampling technique, and the completeness of the subjects' health histories, any direct comparisons of our culture results to the bacterial populations described in these reports should be made with caution.

It is unclear if the *Rhodococcus equi* cultured from one doe represents a true inhabitant or a contaminant, given that the does were housed in close proximity to horses and the organism is soil‐dwelling [54]. Even so, the presence of *Rhodococcus equi* in TTWF from a goat should not be readily discounted if the clinical presentation is supportive of pneumonia. A recent case series of *R. equi* pneumonia in goats identified clinical bronchopneumonia similar to that seen in foals [24]. Most *Rhodococcus* isolates from goats are of the strain containing plasmid gene VapN, and many are nonvirulent [24, 55]. As such, these isolates are unique from the virulent strains containing the VapA plasmid commonly isolated in foals [56]. Susceptibility to the pathogen may be associated with age (and thereby with ability to mount a Th1 response), underlying disease (such as haemonchosis or concurrent respiratory inflammation), or both. *R. equi* appears to have a concurrent proclivity for the liver in goats, and, as such, the liver should be assessed in addition to the airway if *R. equi* is cultured from a goat in which the clinical presentation is supportive [24, 55, 57].

### 4.3 Excluded Samples

The three does excluded for cytologic evidence of infection (characterized by the presence of intracellular bacteria), the cytologic presence of extracellular fungal hyphae (Figure 2) or both represented interesting scenarios. At the time of sampling, all three does were assessed as clinically normal by physical examination, thoracic ultrasound examination, and hematology, and remained clinically healthy in the month after sampling. Their bacterial culture profiles were not unique relative to the remainder of the study population (Table S4), but this is not unusual because commensal bacteria are common in bronchopneumonia in ruminants when conditions are conducive for their overgrowth [58]. Clinical and cytologic evidence was insufficient to establish that fungal hyphae represented colonization or infection. Because fungal elements are ubiquitous in hay and soil, their presence could reflect exposure or a lack of clearance of respiratory secretions from unrelated airway inflammation [59, 60, 61]. These does had the highest TTWF neutrophil counts (72.5%, 56.5%, and 37.25%) in the study, supporting the role of intracellular bacteria as pathogens contributing to inflammation and the presence of fungal hyphae as an effect of inflammation and associated compromised lower airway clearance. Fungal culture was performed for one excluded sample where fungal hyphae were visualized cytologically, but no fungal growth was observed after 14 days. We intended to identify the fungi that grew on four aerobic bacterial culture plates, but were unable to do so because of sample mishandling. Regardless, both the human and veterinary medical literature report poor agreement between fungal culture and fungal detection on cytology of lower airway washes, prompting skepticism in the utility of culture when pursuing a mycologic differential diagnosis [48, 60, 62, 63]. Future research is needed to characterize the normal mycologic population in healthy goats, ascertain the most accurate diagnostic method of identifying fungal airway pathogens in goats, and evaluate the relationship between airway inflammation, positive bacterial culture, and the cytologic presence of fungal elements. The scenarios presented by these three does also emphasize the importance of interpreting TTWF cytology in the context of the clinical presentation and other diagnostic findings.

### 4.4 TTW Procedure Appraisal

The sedation protocol was selected with the intention that: (1) the doe would retain the ability to swallow, thereby mitigating the risk of sample contamination by saliva and associated oropharyngeal cells and bacteria; (2) the doe would remain standing but compliant to allow the procedure to be performed quickly and safely; and (3) the pool of TTWF would not be disrupted by excessive coughing. It is noteworthy that some coughing is likely to be helpful to expectorate more representative samples from the smaller airways [64]. Although some variation in the depth of sedation was noted, the sedation protocol generally produced a minimally reactive state for 5–7 min, which was sufficient to complete the procedure. As expected, less coughing occurred in more markedly sedated animals. If paroxysmal coughing was encountered upon initial penetration of the trachea, coughing improved or resolved within a short time when the irrigating catheter was no longer being advanced. Recovery was rapid and the does were ambulating with minimal to no ataxia 30 min after injection of the midazolam and butorphanol combination.

It was initially observed that the typical aliquot dose used for the TTW procedure in horses (approximately 0.06–0.2 mL/kg) yielded little to no recovery of TTWF [65, 66]. The instillation was increased to 0.4 ± 0.2 mL/kg as the minimum volume range that recovered at least 1 mL of TTWF. Although a large saline aliquot relative to the typical weight‐to‐volume ratio in horses, it was in alignment with a typical ratio for medium‐ to large‐breed dogs [64, 67]. It is common in dogs to recover < 10% of the saline aliquot, also consistent with the average aliquot recovery from our study population [64]. Although no study has directly demonstrated this concept, it is logical that fluid would not pool as readily in tracheas of smaller diameter. Future research evaluating optimization of TTW technique in goats should consider a relationship between tracheal diameter and aliquot recovery volume.

The development of edema and abscessation potentially could have been averted by the creation of a smaller skin incision, the use of an introducer of smaller gauge, and prompt coverage of the site with a bandage left in place for at least 1 h. Although not detected in any animal after the TTW, these procedural modifications also would mitigate the risk of formation of SC emphysema and contamination of the TTW site, especially if the procedure is performed in the field [64]. The 10‐gauge introducer (as part of the Mila Large Animal Transtracheal Wash Kit) was used for all goats to maintain consistency of technique and to demonstrate the versatility of the kit over a wide range of body weights. Placement of the introducer in the smaller does (weighing 20.5–29.5 kg) was more challenging than in the larger does. As such, goats weighing ≤ 20 kg would likely be more suited for the techniques used for medium or large breed dogs where a 14‐gauge or 16‐gauge needle or over‐the‐needle catheter facilitates the passage of a red rubber or polypropylene catheter of smaller diameter [64]. A 1 cm incision was made for ease of entrance into the trachea, and the volume of 2% lidocaine (2 mL) was selected to provide a sufficiently large area of analgesia for the incision. The incision length and 2% lidocaine volume were kept the same for all goats to maintain consistency for post‐procedural complication assessment. Because of the thickness of goat skin, it is our experience that a skin incision is helpful before the insertion of the introducer into the trachea. Passage through the skin will subject the introducer to drag and could result in excess force, compromising precision and safety when entering the trachea. It is conceivable that an incision as small as a full‐thickness stab incision with a #15 surgical blade (accompanied by a decreased volume of 2% lidocaine for regional anesthesia) would be sufficient to facilitate the passage of the introducer into the trachea.

### 4.5 Study Limitations

One limitation of the transtracheal approach is the introduction of blood contamination into the sample because it has the potential to impact cytology results. Variable amounts of blood were seen in all samples, but this contamination was subjectively mild in most cases and did not substantially influence the TTWF leukocyte counts. This finding held true even in the two samples with the most marked blood contamination (> 100 000 RBC/μL) because their neutrophil counts still fell within the established RI (27.5% and 15.75% neutrophils, respectively; Table S2). Additionally, blood contamination was not mistaken for pathologic hemorrhage. Even with up to a 4‐h delay in processing some samples, no erythrophagia or hemosiderin was visualized. Nevertheless, for samples with potentially longer processing delays, it is recommended to include an unstained direct smear of the sample in case of spurious erythrophagia or other storage artifacts [45, 68].

A limitation with regard to characterization of the respiratory microbiome is that bacterial culture is a suboptimal means of detecting the presence of certain microbes in comparison to amplification of genetic material. It is not economically feasible for most small ruminant practitioners to perform PCR to detect every suspected respiratory pathogen. Moreover, bacterial culture enables in vitro antimicrobial susceptibility testing to be performed to guide treatment, consistent with antimicrobial stewardship. Therefore, our study was designed to reflect typical practice, wherein TTWF fluid diagnostic testing consists of cytologic and culture analysis. In our experience, *Mycoplasma* is a commonly encountered respiratory pathogen in goats dwelling in East‐Central Texas that does not readily grow on standard aerobic culture media [69, 70]. Identification of genetic material by PCR typically is performed in combination with culture if there is clinical suspicion for this organism [71]. Including concurrent PCR would have strengthened the study because *Mycoplasma* also can be a normal inhabitant of the respiratory flora in the airway of goats [22, 69, 72].

One final limitation relates to both the size and homogeneity of our cohort. Ideally, for determining de novo RIs, the ASVCP guidelines suggest including > 120 reference individuals [44]. Doing so often is not possible in veterinary studies, but it bears mentioning because using lower numbers limits the available statistical methods for RI determination and contributes to less accuracy and increased uncertainty in the RI [44]. Additionally, our study cohort consisted of only a single herd of Boer does, and thus the results are not necessarily applicable to all goats. Differences in age, breed, sex, vaccine status, pregnancy status, respiratory disease history, non‐respiratory comorbidity, environmental conditions, diet, husbandry technique, and season all have the potential to affect normal TTWF cytologic characteristics [20, 46, 73, 74]. Moreover, differences in sampling technique, aliquot volume, sampling depth, recovered TTWF volume, sample storage, and sample analysis also could impact cytologic and microbiologic results [45, 50, 68, 74]. Given these limitations, RI validation is recommended before adoption of these RIs in different clinical settings [44]. Nonetheless, our findings serve as a useful baseline to guide interpretation of TTWF in goats until more broadly applicable data is available.

## 5 Conclusions

With the growing prevalence of antimicrobial resistance, TTW is a valuable diagnostic tool to guide treatment in goats with lower airway disease. Understanding the cytologic and microbiologic profiles of TTWF in health is vital to discerning the magnitude of deviation in diseased states. The findings from our cohort in East‐Central Texas should be considered when interpreting TTWF in goats, particularly in populations of similar signalment. Albeit inconsistent, several well‐recognized pathogens of the lower airway are present in the TTWF of healthy goats. The culture results should be appraised against both the clinical picture and expectation for an adequately cellular, macrophage‐predominant TTWF cytology, regardless of the cytologic presence of extracellular bacteria. The interactions among airway microorganisms within their microenvironment are complex and dynamic, and future studies are needed to further elucidate the most important influences on TTWF cytology and microbiology results.

## Disclosure

Authors declare no off‐label use of antimicrobials.

## Ethics Statement

Approved by the Texas A&M University Institutional Animal Care and Use Committee and the Clinical Research Review Committee of the Texas A&M College of Veterinary Medicine & Biomedical Sciences (Protocol #2021‐0341). Authors declare human ethics approval was not needed.

## Conflicts of Interest

The authors declare no conflicts of interest.

## Supporting information


**Table S1:** Complete blood count findings from 33 healthy Boer does on which transtracheal wash fluid was obtained.
**Table S2:** Total nucleated cell count with leukocyte differential in transtracheal wash fluid samples from 33 healthy Boer does.
**Table S3:** Cytologic findings in transtracheal wash fluid from 33 healthy Boer does.
**Table S4:** Aerobic and anaerobic culture results of transtracheal wash fluid samples from 33 healthy Boer does.
