# Supplementary material for: Transtracheal Wash Fluid Collection and Analysis in Healthy Goats
Source: J Vet Intern Med. 2025 Sep 17;39(5):e70211. doi: 10.1111/jvim.70211 (PMC12441592; doi:10.1111/jvim.70211)
Supplement: Supplementary file 1 — Table S1: Complete blood count findings from 33 healthy Boer does on which transtracheal wash fluid was obtained. Table S2: Total nucleated cell count with leukocyte differential in transtracheal wash fluid samples from 33 healthy Boer does. Table S3: Cytologic findings in transtracheal wash fluid from 33 healthy Boer does. Table S4: Aerobic and anaerobic culture results of transtracheal wash fluid samples from 33 healthy Boer does. [file JVIM-39-e70211-s001.docx]

**Supplemental Table 1: Complete blood count findings from 33 healthy Boer does on which transtracheal wash fluid was obtained.**

| ID | Tot WBCs (x10^3/µL) | | Abs Neut (x10^3/µL) | Abs Lymph (x10^3/µL) | Abs Mono (x10^3/µL) | Abs Eos (x10^3/µL) | Abs Baso (x10^3/µL) | HCT (%) | Plasma Protein (g/dL) | Fibrinogen (mg/dL) | WBC Morph |
| --- | --- | --- | --- | --- | --- | --- | --- | --- | --- | --- | --- |
| 1 | | 20.17 | 6.05 | 13.51 | 0 | 0.61 | 0 | 31 | 6 | 200 | Normal |
| 2 | | 11.72 | 6.09 | 5.63 | 0 | 0 | 0 | 31.1 | 7 | 100 | Normal |
| 3 | | 7.67 | 4.68 | 2.76 | 0.08 | 0.15 | 0 | 31.4 | 6.5 | 200 | Normal |
| 4 | | 13.97 | 8.1 | 5.17 | 0.42 | 0.28 | 0 | 27.8 | 5.9 | 300 | Reactive lymph |
| 5 | | 17.37 | 8.51 | 7.99 | 0.17 | 0.52 | 0.17 | 32.4 | 5.7 | 300 | Normal |
| 6 | | 13.32 | 8.92 | 3.86 | 0.4 | 0 | 0.13 | 26.1 | 6.2 | 200 | Normal |
| 7 | | 14.13 | 4.66 | 9.04 | 0.28 | 0 | 0.14 | 30.7 | 6.5 | 200 | Normal |
| 8 | | 12.87 | 6.82 | 4.76 | 0 | 0.9 | 0.39 | 22.5 | 6.6 | 200 | Normal |
| 9 | | 12.47 | 6.73 | 5.49 | 0 | 0.12 | 0.12 | 28.3 | 6.9 | 300 | Reactive lymph |
| 10 | | 10.05 | 6.33 | 3.32 | 0.3 | 0 | 0.1 | 29.1 | 6.3 | 300 | Normal |
| 11 | | 13.82 | 7.88 | 5.25 | 0.14 | 0.14 | 0.41 | 26.6 | 6.6 | 200 | Normal |
| 12 | | 13.8 | 6.35 | 6.76 | 0.14 | 0.55 | 0 | 27.7 | 7 | 100 | Normal |
| 13 | | 10.86 | 6.95 | 3.69 | 0 | 0.11 | 0.11 | 32.2 | 7.3 | 100 | Normal |
| 14 | | N/I |  |  |  |  |  |  |  |  |  |
| 15 | | 7.53 | 2.71 | 4.67 | 0.08 | 0.08 | 0 | 30.7 | 6.8 | 200 | Normal |
| 16 | | 12.02 | 5.41 | 5.41 | 0.36 | 0.72 | 0.12 | 29.8 | 6.7 | 200 | Normal |
| 17 | | 12.91 | 5.42 | 6.46 | 0.13 | 0.77 | 0.13 | 29.3 | 7.1 | 200 | Normal |
| 18 | | 10.17 | 4.98 | 4.78 | 0.2 | 0.1 | 0.1 | 29.5 | 7.5 | 300 | Normal |
| 19 | | 11.47 | 5.51 | 5.62 | 0 | 0.23 | 0.11 | 27.1 | 7.2 | 200 | Normal |
| 20 | | 8.86 | 3.99 | 4.52 | 0.18 | 0.18 | 0 | 25.9 | 6.7 | 200 | Normal |
| 21 | | 14.91 | 7.75 | 6.26 | 0.15 | 0.45 | 0.3 | 26.9 | 7 | 200 | Normal |
| 22 | | 7.54 | 2.04 | 5.13 | 0.15 | 0.15 | 0.08 | 28.3 | 7.3 | 100 | Normal |
| 23 | | 8.78 | 3.69 | 4.74 | 0.09 | 0.18 | 0.09 | 31.2 | 7.7 | 500 | Normal |
| 24 | | 9.32 | 4.94 | 4.38 | 0 | 0 | 0 | 27.8 | 7.6 | 200 | Reactive lymph |
| 25 | | 9.43 | 3.58 | 4.53 | 0.28 | 0.94 | 0.09 | 31.4 | 7.1 | 200 | Normal |
| 26 | | N/I |  |  |  |  |  |  |  |  |  |
| 27 | | N/I |  |  |  |  |  |  |  |  |  |
| 28 | | 12.87 | 6.82 | 4.76 | 0 | 0.9 | 0.39 | 22.5 | 6.6 | 200 | Normal |
| 29 | | N/I |  |  |  |  |  |  |  |  |  |
| 30A Excluded | | 7.48 | 3.81 | 3.29 | 0 | 0.22 | 0.15 | 28.5 | 7.5 | 300 | Reactive lymph |
| 31A Excluded | | 6.76 | 2.64 | 3.65 | 0 | 0.41 | 0.07 | 31 | 7.4 | 200 | Reactive lymph |
| 32A Excluded | | 8.48 | 4.75 | 3.48 | 0.08 | 0 | 0.17 | 30.8 | 6 | 200 | Normal |
| 33A Excluded | | 13.33 | 4.13 | 7.20 | 0.27 | 1.73 | 0 | 29.2 | 7.4 | 300 | Normal |

Abbreviations: Tot, total; WBC, white blood cells; Abs, absolute; Neut, neutrophils; Lymph, lymphocytes; Mono, monocytes; Eos, eosinophils; Baso, basophils; HCT, hematocrit; Morph, morphology; N/I, not interpretable

**Supplemental Table 2: Total nucleated cell count with leukocyte differential in transtracheal wash fluid samples from 33 healthy Boer does.**

| ID | TNC | RBC (tot) | Neut (tot) | Lymph (tot) | Mφ (tot) | Eos (tot) | Mast (tot) | Neut (%) | Lymph (%) | Mφ (%) | Eos (%) | Mast (%) |
| --- | --- | --- | --- | --- | --- | --- | --- | --- | --- | --- | --- | --- |
| 1 | 3902 | 5350 | 91 | 45 | 264 | 0 | 0 | 22.75 | 11.25 | 66.00 | 0.00 | 0.00 |
| 2 | 5434 | 478453 | 110 | 127 | 160 | 3 | 0 | 27.50 | 31.75 | 40.00 | 0.75 | 0.00 |
| 3 | 3519 | 6864 | 49 | 81 | 270 | 0 | 0 | 12.25 | 20.25 | 67.50 | 0.00 | 0.00 |
| 4 | 1806 | 275289 | 63 | 50 | 282 | 5 | 0 | 15.75 | 12.50 | 70.50 | 1.25 | 0.00 |
| 5 | 359 | 519 | 46 | 157 | 195 | 2 | 0 | 11.50 | 39.25 | 48.75 | 0.50 | 0.00 |
| 6 | 1860 | 1563 | 45 | 44 | 310 | 1 | 0 | 11.25 | 11.00 | 77.50 | 0.25 | 0.00 |
| 7 | 8827 | 22393 | 129 | 42 | 229 | 0 | 0 | 32.25 | 10.50 | 57.25 | 0.00 | 0.00 |
| 8 | 3473 | 22028 | 75 | 35 | 286 | 4 | 0 | 18.75 | 8.75 | 71.50 | 1.00 | 0.00 |
| 9 | 1358 | 12546 | 57 | 29 | 314 | 0 | 0 | 14.25 | 7.25 | 78.50 | 0.00 | 0.00 |
| 10 | 464 | 1633 | 35 | 72 | 292 | 1 | 0 | 8.75 | 18.00 | 73.00 | 0.25 | 0.00 |
| 11 | 684 | 715 | 15 | 31 | 354 | 0 | 0 | 3.75 | 7.75 | 88.50 | 0.00 | 0.00 |
| 12 | 2917 | 2742 | 25 | 15 | 360 | 0 | 0 | 6.25 | 3.75 | 90.00 | 0.00 | 0.00 |
| 13 | 470 | 20819 | 61 | 97 | 241 | 1 | 0 | 15.25 | 24.25 | 60.25 | 0.25 | 0.00 |
| 14 | 1617 | 512 | 43 | 47 | 308 | 2 | 0 | 10.75 | 11.75 | 77.00 | 0.50 | 0.00 |
| 15 | 4773 | 71264 | 10 | 62 | 327 | 1 | 0 | 2.50 | 15.50 | 81.75 | 0.25 | 0.00 |
| 16 | 1217 | 1942 | 40 | 19 | 341 | 0 | 0 | 10.00 | 4.75 | 85.25 | 0.00 | 0.00 |
| 17 | 1150 | 28140 | 15 | 18 | 367 | 0 | 0 | 3.75 | 4.50 | 91.75 | 0.00 | 0.00 |
| 18 | 1974 | 3335 | 48 | 19 | 332 | 1 | 0 | 12.00 | 4.75 | 83.00 | 0.25 | 0.00 |
| 19 | 783 | 6548 | 70 | 69 | 249 | 4 | 8 | 17.50 | 17.25 | 62.25 | 1.00 | 2.00 |
| 20 | 984 | 1632 | 31 | 15 | 354 | 0 | 0 | 7.75 | 3.75 | 88.50 | 0.00 | 0.00 |
| 21 | 1927 | 18556 | 92 | 57 | 251 | 0 | 0 | 23.00 | 14.25 | 62.75 | 0.00 | 0.00 |
| 22 | 1255 | 16017 | 18 | 32 | 350 | 0 | 0 | 4.50 | 8.00 | 87.50 | 0.00 | 0.00 |
| 23 | 1407 | 9612 | 82 | 33 | 283 | 1 | 1 | 20.50 | 8.25 | 70.75 | 0.25 | 0.25 |
| 24 | 3423 | 4364 | 63 | 16 | 321 | 0 | 0 | 15.75 | 4.00 | 80.25 | 0.00 | 0.00 |
| 25 | 1609 | 7239 | 49 | 12 | 338 | 0 | 1 | 12.25 | 3.00 | 84.50 | 0.00 | 0.25 |
| 26 | 1404 | 7878 | 42 | 43 | 314 | 1 | 0 | 10.50 | 10.75 | 78.50 | 0.25 | 0.00 |
| 27 | 2322 | 12337 | 84 | 41 | 272 | 2 | 1 | 21.00 | 10.25 | 68.00 | 0.50 | 0.25 |
| 28 | 2142 | 20728 | 61 | 47 | 275 | 4 | 13 | 15.25 | 11.75 | 68.75 | 1.00 | 3.25 |
| 29 | 1235 | 10781 | 64 | 58 | 278 | 0 | 0 | 16.00 | 14.50 | 69.50 | 0.00 | 0.00 |
| 30A Excluded | 2649 | 80729 | 290 | 23 | 73 | 11 | 3 | 72.50 | 5.75 | 18.25 | 2.75 | 0.75 |
| 31A Excluded | 4770 | 11286 | 149 | 16 | 235 | 0 | 0 | 37.25 | 4.00 | 58.75 | 0.00 | 0.00 |
| 32A Excluded | 3946 | 8475 | 226 | 3 | 171 | 0 | 0 | 56.50 | 0.75 | 42.75 | 0.00 | 0.00 |
| 33A Excluded | Low cellularity | N/A | N/A | N/A | N/A | N/A | N/A | N/A | N/A | N/A | N/A | N/A |

Abbreviations: TNC, total nucleated cells; Tot, total; RBC, red blood cells; Neut, neutrophils; Lymph, lymphocytes; Mφ, macrophages; Eos, eosinophils; Mast, mast cells; N/A, not applicable

**Supplemental Table 3: Cytologic findings in transtracheal wash fluid from 33 healthy Boer does.**

| ID | Epi Sheets | Environ Inhal | BiMφ | MNGC | Leukophagia | Bacteria | Other |
| --- | --- | --- | --- | --- | --- | --- | --- |
| 1 | few | present | present | absent | absent | none |  |
| 2 | few | present | present | present | present | none |  |
| 3 | few | present | present | present | present | none | rare plasma cells |
| 4 | many | present | present | absent | present | none |  |
| 5 | many | absent | present | absent | present | none |  |
| 6 | few | present | present | present | present | none |  |
| 7 | none | present | present | absent | present | none |  |
| 8 | few | present | present | absent | present | none | few squamous cells |
| 9 | few | absent | present | present | present | none |  |
| 10 | few | present | absent | absent | absent | none |  |
| 11 | few | absent | present | absent | absent | none |  |
| 12 | few | present | present | absent | absent | rare extracell cocci |  |
| 13 | many | absent | absent | absent | present | rare extracell cocci |  |
| 14 | many | present | present | absent | present | rare extracell cocci |  |
| 15 | many | absent | present | present | present | none |  |
| 16 | few | present | absent | absent | absent | rare extracell rod |  |
| 17 | few | present | present | present | present | rare extracell cocci |  |
| 18 | mod | present | present | present | present | rare extracell rod chains |  |
| 19 | few | present | absent | absent | present | none | rare squamous cells |
| 20 | many | present | present | absent | absent | none |  |
| 21 | many | present | present | absent | absent | none |  |
| 22 | many | present | present | present | absent | none |  |
| 23 | mod | present | present | present | present | rare mixed extracell |  |
| 24 | few | present | present | present | present | rare extracell cocci/rods | necrotic epithelium |
| 25 | few | present | present | absent | present | none |  |
| 26 | few | present | present | absent | absent | rare extracell cocci |  |
| 27 | many | present | present | present | present | none |  |
| 28 | few | present | present | absent | absent | none |  |
| 29 | many | absent | present | absent | absent | none |  |
| 30A Excluded | mod | present | absent | absent | present | many intracell rods | branching fungal hyphae |
| 31A Excluded | mod | present | present | present | absent | rare mixed extracell | rare branching fungal hyphae |
| 32A Excluded | few | present | present | absent | present | mod extracell rod, rare intracell rod |  |
| 33A Excluded | N/A | N/A | N/A | N/A | N/A | N/A |  |

Abbreviations: Epi Sheets, epithelial sheets; Environ Inhal, environmental inhalants; BiMφ, binucleated macrophages; MNGC, multinucleated giant cells; Extracell, extracellular; intracell, intracellular; Mod, moderate; N/A, not applicable

**Supplemental Table 4: Aerobic and anaerobic culture results of transtracheal wash fluid samples from 33 healthy Boer does.**

| ID | Aerobic and Anaerobic Culture | Fungal Growth |
| --- | --- | --- |
| 1 | Mixed bacterial growth | Yes |
| 2 | Mixed bacterial growth |  |
| 3 | *B. trehalosi*, *F. necrophorum*, Mixed bacterial growth |  |
| 4 | *Enterobacter* sp. | Yes |
| 5 | Mixed bacterial growth |  |
| 6 | *R. equi* |  |
| 7 | *B. trehalosi* |  |
| 8 | *B. trehalosi*, *Fusobacterium* sp., *T. pyogenes*, Mixed bacterial growth |  |
| 9 | *Mannheimia* sp., Mixed bacterial growth |  |
| 10 | No bacteria isolated |  |
| 11 | Mixed bacterial growth |  |
| 12 | *E. coli* |  |
| 13 | Mixed bacterial growth |  |
| 14 | No bacteria isolated |  |
| 15 | No bacteria isolated |  |
| 16 | No bacteria isolated | Yes |
| 17 | Mixed bacterial growth |  |
| 18 | *Enterobacter* sp., Mixed bacterial growth |  |
| 19 | Mixed bacterial growth |  |
| 20 | Mixed bacterial growth |  |
| 21 | *Enterobacter* sp. |  |
| 22 | Mixed bacterial growth |  |
| 23 | Mixed bacterial growth |  |
| 24 | Mixed bacterial growth |  |
| 25 | Mixed bacterial growth |  |
| 26 | *B. trehalosi* |  |
| 27 | *B. trehalosi*, *Mannheimia* sp., Mixed bacterial growth |  |
| 28 | Mixed bacterial growth | Yes |
| 29 | No bacteria isolated |  |
| 30A Excluded | Mixed bacterial growth |  |
| 31A Excluded | *E. coli*, Mixed bacterial growth | No |
| 32A Excluded | Mixed bacterial growth |  |
| 33A Excluded | *Enterobacter* sp., Mixed bacterial growth |  |
